# Linezolid-Resistant *Staphylococcus epidermidis,* Portugal, 2012

**DOI:** 10.3201/eid2005.130783

**Published:** 2014-05

**Authors:** Mariana Barros, Raquel Branquinho, Filipa Grosso, Luísa Peixe, Carla Novais

**Affiliations:** Faculdade de Farmácia da Universidade do Porto, Porto, Portugal

**Keywords:** Staphylococcus epidermidis, linezolid resistance, biofilm, outbreak, 23S rRNA mutations, L3 protein mutations, bacteria

**To the Editor:** Linezolid is a therapeutic option for skin and soft tissue infections and pneumonia caused by multidrug-resistant gram-positive bacteria (e.g., *Staphylococcus* spp.), which occur at higher rates in Portugal than in other European countries (www.ecdc.europa.eu/en/publications/Publications/annual-epidemiological-report-2013.pdf). *Staphylococcus epidermidis* are skin and mucosal commensal bacteria; infections in humans are mostly linked to indwelling medical devices. The ability of *S. epidermidis* to acquire resistance to antimicrobial drugs and to produce biofilm can seriously compromise the success of therapy; in many institutions worldwide, rates of methicillin resistance are >70% (*1*). Rates of *S. epidermidis* linezolid resistance on various continents have been low and are associated with mutations in the central loop of 23S rRNA V domain or ribosomal proteins (L3, L4, and L22) and with acquisition of the *cfr* gene, which codifies for ribosomal methyltransferase (*1*–*3*). 

To our knowledge, in Portugal only 1 linezolid-resistant *S. epidermidis* isolate, from a dog with severe otitis, has been described (*4*). We report nosocomial emergence of methicillin- and linezolid-resistant *S. epidermidis* in Portugal.

We characterized 5 linezolid-resistant *Staphylococcus* isolates recovered during May–November 2012 from blood and catheters of patients in 4 wards of a 362-bed hospital in central Portugal. The origin of 1 isolate is unknown. Epidemiologic features are described in the Table. The patients had received linezolid during the present (n = 2 patients) or previous (n = 2 patients) hospitalizations, suggesting that the latter 2 patients could have been colonized with linezolid-resistant strains when discharged from the first hospitalization. Information about receipt of linezolid was not available for 1 patient.

*S. epidermidis* was identified by using a Vitek II system (bioMérieux, Marcy L’Étoile, France), and susceptibility to antimicrobial drugs was studied by using agar dilution (linezolid, vancomycin) or disk diffusion (another 10 drugs; Table) (*5*). All isolates were screened by PCR and sequenced for the *cfr* gene and for mutations in the 23S rRNA V domain and in genes (*rplC, rplD* and *rplV*) encoding the L3, L4, and L22 ribosomal proteins (*6*–*8*). Clonal relatedness was determined by pulsed-field gel electrophoresis (macrorestriction with *Sma*I) and by multilocus sequence typing (www.cdc.gov/hai/pdfs/labsettings/ar_mras_pfge_s_aureus.pdf and http://sepidermidis.mlst.net). *S. epidermidis* from patient 1 was searched for in vitro adherence to abiotic surfaces by using a biomass quantification assay (*9*) and strain ICE9 as a positive control. 

All *S. epidermidis* isolates were resistant to multiple drugs, including linezolid (MIC>32 mg/L), cefoxitin, chloramphenicol, cotrimoxazole, ciprofloxacin, clindamycin, and aminoglycosides, and susceptible to only 4 drugs tested, including vancomycin (MIC = 2 mg/L) (Table). To characterize linezolid resistance, we compared the study isolates with linezolid-susceptible *S. epidermidis* RP62A/American Type Culture Collection 35984 sequence (GenBank accession no. CP000029). Study isolates contained the mutations T2530A, T2504A, and G2631T, although T2504A and G2631T were also present in linezolid-susceptible *S. epidermidis* RP62A (*1*,*2*). The most commonly reported G2576T mutation was not detected (*1*,*2*). We also compared study isolates with *S. epidermidis* RP62A and observed nucleotide mutations consistent with L94V (L101V from *S. epidermidis* American Type Culture Collection 12228, not associated with linezolid resistance) and G152D amino acid changes (*2*,*8*) and amino acid changes in the new D159E and A160P in L3 ribosomal protein. Mutations in this protein were linked to linezolid resistance, although definitive conclusions are not available (*8*). The *cfr* gene and mutations in ribosomal proteins L4 or L22 were not detected in the study isolates.

**Table:** Epidemiologic features and antimicrobial drug resistance of linezolid -resistant *Staphylococcus epidermidis* isolates from a hospital, Portugal, 2012*

Characteristic	Patient no.†				
	1	2	3	4	5
Epidemiologic features					
Date of isolation	2012 May 8	2012 Aug 7	2012 Oct 23	2012 Nov 7	2012 Nov 11
Hospital ward	Men’s surgery	Unknown	Medicine I	Emergency unit‡	Emergency unit‡
Pathology	Gastric neoplasia§¶	Unknown	Multiple§	Acute lung edema	Multiple
Clinical sample	Catheter	Blood	Catheter	Blood	Blood
Patient sex/age, y	M/75	Unknown	F/87	M/78	M/87
Previous linezolid	Yes	Unknown	Yes#	Yes‡	Yes‡
PFGE type	A	A	A	A	A
Sequence type	2**				
Biofilm production (OD_570nm_)	Strong (2.33 ± 0.34)**††				
Drug resistance					
Linezolid (MIC, mg/L)	R (32)	R (32)	R (32)	R (32)	R (32)
Vancomycin (MIC, mg/L)	S (2)	S (2)	S (2)	S (2)	S (2)
Cefoxitin	R	R	R	R	R
Gentamicin	R	R	R	R	R
Tobramycin	R	R	R	R	R
Ciprofloxacin	R	R	R	R	R
Clindamycin	R	R	R	R	R
Erythromycin	S	I	S	I	S
Quinupristin–dalfopristin	S	S	S	S	S
Chloramphenicol	R	R	R	R	R
Tetracycline	S	S	S	S	S
Cotrimoxazole	R	R	R	R	R
Molecular features					
*cfr* gene	–	–	–	–	–
23S rRNA mutations					
T2504A	+	+	+	+	+
G2631T	+	+	+	+	+
T2530A	+	+	+	+	+
L3 ribosomal protein mutations					
Leu94Val	+**				
Gly152Asp	+**				
Asp159Glu	+**				
Ala160Pro	+**				
L4 or L22 ribosomal protein mutations	None				

*PFGE, pulsed-field gel electrophoresis; OD, optical density; R, resistant; S, susceptible; I, Intermediate resistance; –, negative; +, positive. Blank cells indicate not tested. †A sixth linezolid-resistant *S. epidermidis* isolate was detected in December 2012; however, access to this isolate was not possible during this study. ‡Patients 4 and 5 were hospitalized in Medicine II a month before linezolid-resistant *S. epidermidis* was isolated. Therapy with linezolid was started during this first hospitalization. For patient 4, duration of linezolid therapy was at least 12 d. For patient 5, duration of therapy is unknown. §Long-stay hospitalization. ¶Followed up in oncology ward since 2011. #Patient 3 received linezolid for 11 d before linezolid-resistant *S. epidermidis* was detected. **Studied in S. *epidermidis* from patient 1 only, representative isolate of the PFGE type A. ††For the interpretation of the results, the cutoff optical density (ODc) was defined as 3 SDs above the mean OD of the negative control (culture medium). Strains were classified as nonadherent (OD<ODc), weakly adherent (ODc<OD<2×ODc), moderately adherent (2xODc<OD<4×ODc), or strongly adherent (4×ODc<OD).

All isolates recovered had the same pulsed-field gel electrophoresis type and belonged to sequence type (ST) 2/clonal complex (CC) 5 (ST2 formerly belonged to CC2) (*10*) also detected among linezolid-resistant *S. epidermidis* from Europe, Brazil, and the United States (*1*–*3*). *S. epidermidis* from patient 1, considered representative of the observed clone, revealed a high ability to adhere to abiotic surfaces and grow in the biofilm form, which can facilitate infections associated with indwelling medical devices. This strain was classified as strongly adherent and had higher optical density (OD_570nm_ = 2.33 ± 0.34) than a blank sample (culture medium: Luria Bertani broth + glucose; OD_570nm_ = 0.2 ± 0.03). The OD_570nm_ of the positive-control was 2.69 ± 0.44.

*S. epidermidis* ST2/CC5 is disseminated in hospital settings worldwide and is characterized by a high level of genetic diversity, an increased recombination/mutation rate, biofilm production ability, and acquisition of a high number of staphylococcal cassette chromosome *mec* elements (*10*). In Portugal, *S. epidermidis* ST2/CC5 has been observed in the community (*10*). We report emergence of methicillin- and linezolid-resistant *S. epidermidis* in a hospital in Portugal and its persistence for at least 7 months. Identification of the successful multidrug-resistant *S. epidermidis* ST2/CC5 clonal lineage highlights the need for strict infection control procedures and revision of therapeutic strategies (e.g., linezolid use for the treatment of methicillin-resistant *Staphylococcus* spp. only when vancomycin is not a treatment option because of elevated MIC or clinical failures) to preserve therapeutic effectiveness of linezolid. Effective control of linezolid-resistant *S. epidermidis*, including among hospital-discharged patients who had received linezolid, is critical for preventing the potential for an epidemic in this hospital, and, on a larger scale, in Portugal, as has occurred for other gram-positive methicillin-resistant *S. aureus* and vancomycin-resistant enterococci.

## References

[1] Mendes RE, Deshpande LM, Costello AJ, Farrell DJ. Molecular epidemiology of *Staphylococcus epidermidis* clinical isolates from U.S. hospitals. Antimicrob Agents Chemother. 2012;56:4656–61 . 10.1128/AAC.00279-12 22687512 PMC3421855

[2] de Almeida LM, Lincopan N, Araújo MR, Mamizuka EM. Dissemination of the linezolid-resistant *Staphylococcus epidermidis* clone ST2 exhibiting the G2576T mutation in the 23S rRNA gene in a tertiary-care hospital, Brazil. J Antimicrob Chemother. 2012;67:768–9. 10.1093/jac/dkr538 22186879

[3] Liakopoulos A, Spiliopoulou I, Damani A, Kanellopoulou M, Schoina S, Papafragas E, Dissemination of two international linezolid-resistant *Staphylococcus epidermidis* clones in Greek hospitals. J Antimicrob Chemother. 2010;65:1070–1 . 10.1093/jac/dkq065 20207718

[4] Seixas R, Monteiro V, Carneiro C, Vilela CL, Oliveira M. First report of a linezolid-resistant MRSA (methicillin resistant *Staphylococcus aureus*) isolated from a dog with a severe bilateral otitis in Portugal. Revista Veterinaria. 2011;22:81–4.

[5] Clinical and Laboratory Standards Institute. Performance standards for antimicrobial susceptibility testing: twentieth informational supplement M100–S21. Wayne (PA): The Institute; 2010.

[6] Kehrenberg C, Schwarz S. Distribution of florfenicol resistance genes *fex*A and *cfr* among chloramphenicol-resistant S*taphylococcus* isolates. Antimicrob Agents Chemother. 2006;50:1156–63. 10.1128/AAC.50.4.1156-1163.2006 16569824 PMC1426988

[7] Toh SM, Xiong L, Arias CA, Villegas MV, Lolans K, Quinn J, Acquisition of a natural resistance gene renders a clinical strain of methicillin-resistant *Staphylococcus aureus* resistant to the synthetic antibiotic linezolid. Mol Microbiol. 2007;64:1506–14. 10.1111/j.1365-2958.2007.05744.x 17555436 PMC2711439

[8] Locke JB, Hilgers M, Shaw KJ. Novel ribosomal mutations in *Staphylococcus aureus* strains identified through selection with the oxazolidinones linezolid and torezolid (TR-700). Antimicrob Agents Chemother. 2009;53:5265–74. 10.1128/AAC.00871-09 19752277 PMC2786364

[9] Novais A, Pires J, Ferreira H, Costa L, Montenegro C, Vuotto C, Characterization of globally spread *Escherichia coli* ST131 isolates (1991 to 2010). Antimicrob Agents Chemother. 2012;56:3973–6. 10.1128/AAC.00475-12 22491693 PMC3393462

[10] Rolo J, Lencastre H, Miragaia M. Strategies of adaptation of *Staphylococcus epidermidis* to hospital and community: amplification and diversification of *SCC*mec. J Antimicrob Chemother. 2012;67:1333–41. 10.1093/jac/dks068 22422509

